# Supplementary material for: Polyketide synthase gene clusters in the ericoid mycorrhizal fungus Oidiodendron maius: genome-wide prediction, expression profiling, and functional characterization of knockout mutants
Source: Front Plant Sci. 2026 Sep 16;17:1924430. doi: 10.3389/fpls.2026.1924430 (PMC13623903; doi:10.3389/fpls.2026.1924430)
Supplement: Supplementary file 1 [file SupplementaryFile1.zip › Supplementary Materials/Supplementary Folder 1.zip!/Oidma1_AssemblyScaffolds_Repeatmasked/knownclusterblast/cluster12/jgi.p_Oidma1_39553_mibig_hits.html]

| MIBiG Protein | Description | MIBiG Cluster | MiBiG Product | % ID | % Coverage | BLAST Score | E-value |
| --- | --- | --- | --- | --- | --- | --- | --- |
| CDM36724.1 | Cytochrome\_P450 | BGC0001360 | Mycophenolic\_acid\_biosynthetic\_gene\_cluster | 50 | 97.1428571429 | 369 | 6e-102 |
| ADY00132.1 | putative\_metallo-beta-lactamase\_superfamily\_II\_enzyme | BGC0000104 | Mycophenolic\_acid\_biosynthetic\_gene\_cluster | 48 | 76.3636363636 | 273 | 3.4e-73 |
